# Innate immunogenetic synergy between KIR and Neanderthal-derived *OAS* variants predicts COVID-19 outcomes

**DOI:** 10.1371/journal.pone.0345137

**Published:** 2026-05-27

**Authors:** Hasan Yalim Akin, Timur Tuncali, Emine Begum Gencer, Guldane Cengiz Seval, Elif Mukime Saricaoglu, Ezgi Gulten, Irem Akdemir Kalkan, Gule Cinar, Osman Memikoglu, Ridvan Goksel Anliacik, Ezgi Anliacik, Ergun Karaagaoglu, Klara Dalva, Meral Beksac

**Affiliations:** 1 Cord Blood Bank, Ankara University Faculty of Medicine, Ankara, Turkey; 2 Department of Medical Genetics, Ankara University Faculty of Medicine, Ankara, Turkey; 3 Department of Hematology, Ankara University Faculty of Medicine, Ankara, Turkey; 4 Department of Infectious Diseases and Clinical Microbiology, Ankara University Faculty of Medicine, Ankara, Turkey; 5 Department of Hematology, Immunogenetics Laboratory, Ankara University Faculty of Medicine, Ankara, Turkey; 6 Department of Biostatistics, Lokman Hekim University Faculty of Medicine, Ankara, Turkey; Huaqiao University - Quanzhou Campus: Huaqiao University, CHINA

## Abstract

**Background:**

Genetic factors modulate the progression of coronavirus disease 2019 (COVID-19), but interactions between innate immune gene variants remain incompletely understood. Prior work identified Killer Immunoglobulin-like Receptor (KIR) genotypes and Neanderthal-introgressed *OAS*1/2/3 haplotypes as key modulators of disease severity. Given these parallel findings, we aimed to evaluate the synergistic impact of KIR motifs, Neanderthal-inherited *OAS*1/2/3 variants, and other immune-related SNPs on COVID-19 symptomatology and severity.

**Methods:**

In a cohort of 175 unvaccinated severe acute respiratory syndrome coronavirus 2 (SARS-CoV-2) positive individuals (65 asymptomatic, 47 mild-intermediate, 63 severe), we genotyped KIR/KIR-ligand motifs and variants in *OAS*1/2/3, *IFITM3*, *DPP4*, *TLR7*, and *APOE*. Multivariate logistic regression models assessed independent and interactive predictors of symptomatic and severe disease, and ROC curve analysis was performed to quantify their discriminative ability.

**Results:**

An interaction between the absence of both the protective tAB1/wL KIR motif and the Neanderthal-derived *OAS*1/2/3 alleles significantly predicted symptomatic infection (OR 3.47, P = 0.006) and severe disease (OR 2.41, P = 0.038), achieving overall classification accuracies of 75.4% and 78.9%, respectively. Independent predictors included age, male gender, rs12252 (*IFITM3*) and rs3788979 (*DPP4*) as risk factors, and blood group A and *APOE* ε3ε3 status as protective factors. No rare *TLR7* variants were detected.

**Conclusion:**

This study identifies, for the first time, a synergistic interaction between KIR motifs and Neanderthal-derived *OAS*1/2/3 variants influencing COVID-19 outcomes. These findings highlight the interplay between ancient and modern innate immune adaptations in shaping viral disease susceptibility. Future studies in larger and diverse populations are warranted to validate these immunogenetic interactions.

## Introduction

The clinical course of severe acute respiratory syndrome coronavirus 2 (SARS-CoV-2) infection displays wide heterogeneity, ranging from asymptomatic to critical illness and death [1]. While demographic and clinical factors such as age, comorbidities, and blood group influence coronavirus disease 2019 (COVID-19) severity, host genetics may also play a substantial role in disease outcomes [2–4].

Among immunogenetic factors, Killer Immunoglobulin-like Receptors (KIRs), encoded on chromosome 19q13.4, have been implicated in modulating antiviral responses. Anthropologically KIR genes, which mostly interact with specific human leukocyte antigen (HLA) class I ligands, evolved relatively recently compared to HLA loci and exhibit extensive polymorphism and copy number variations. Previous studies, including our own, have associated specific KIR genotypes and haplotype motifs with COVID-19 severity, highlighting the importance of innate immune signaling diversity, particularly its role in the clearance of SARS-CoV-2 infected cells through NK cell activation mediated by KIR-HLA ligand interactions [5–7].

Another evolutionary aspect of immune responsiveness involves Neanderthal-introgressed genomic regions. Zeberg and Pääbo (2020) identified a Neanderthal-derived haplotype on chromosome 3p21.31, encompassing genes such as *SLC6A20*, *LZTFL1*, and *CCR9*, that confers increased risk of severe COVID-19 [8]. However, a subsequent study by the same group [9] revealed a distinct Neanderthal-inherited haplotype at the *OAS*1/2/3 locus on chromosome 12q24.13, which instead provides protection against severe disease. These findings underscore the dualistic impact of Neanderthal ancestry on COVID-19 outcomes, with both risk-enhancing and protective genomic segments persisting in modern human populations.

The *OAS*1/2/3 gene cluster encodes oligoadenylate synthetases that activate RNase L-mediated degradation of viral RNA, constituting a key arm of the interferon-stimulated antiviral response. Functional studies have shown that the Neanderthal-derived allele at *OAS*1 (rs10774671-G), which preserves an ancestral splice variant, results in higher enzymatic activity and enhanced viral restriction [10,11]. In addition to rs10774671, the Neanderthal-introgressed *OAS* haplotypes include three missense variants (rs2660, rs1293767 and rs1859330) and two synonymous variants (rs1859329 and rs2285932), all of which have been associated with an altered course of various viral infections [9,12]. Considering that the protective Neanderthal alleles at *OAS*1/2/3 are annotated as the reference alleles in GRCh38 (*OAS*1: rs10774671-G and rs2660-G; *OAS*2: rs1293767-C; *OAS*3: rs1859330-G, rs1859329-C and rs2285932-T), individuals lacking the alternate variants in *OAS*1/2/3 are expected to mount more effective innate immune responses to RNA viruses such as SARS-CoV-2.

Given these parallel findings on KIR variability and Neanderthal-introgressed *OAS*1/2/3 alleles, we hypothesized that their interaction might synergistically modulate COVID-19 outcomes. In the present study, we systematically evaluated the combined influence of KIR haplotype motifs, *OAS*1/2/3 protective variants, and additional polymorphisms in immune-related genes (*IFITM3*: restriction of virus replication [13]; *DPP4*: viral entry, inflammatory response and insulin resistance [14]; *TLR7*: interferon induction [15]; *APOE*: immunometabolic regulation [16]) on COVID-19 symptomatology and severity. Notably, individuals were classified as *OAS*1/2/3(-) if they carried at least one Neanderthal-derived protective allele (rs10774671-G, rs2660-G, rs1293767-C, rs1859330-G, rs1859329-C or rs2285932-T), consistent with prior functional and population genetic evidence. By integrating these immunogenetic factors within multivariate predictive models, we sought to identify key genetic determinants of disease progression in a cohort of unvaccinated individuals infected with SARS-CoV-2.

## Methods

### Participants

This study was approved by the Ministry of Health and Ethical Committee of Ankara University (Ministry of Health, 05.05.2021; Local Ethical Committee, i5-266–20 and i6-420–21) with a planned study design. This study included a total of 175 unvaccinated patients (65 asymptomatic, 47 mild-intermediate and 63 severe) who tested positive for COVID-19 by PCR and had a self-reported history of their first confirmed infection; of whom 104 of the patients (52 asymptomatic, 27 mild-intermediate and 25 severe) were included from our previous study [5] during the first surge of COVID-19 in Turkey between 6 June 2020–1 November 2020. In order to reach an adequate sample size for extended analysis of KIRs and SNPs together, additional 71 patients (13 asymptomatic, 20 mild-intermediate and 38 severe) were recruited into the study between 11 July 2021–08 December 2021. All asymptomatic patients were tested for SARS-CoV-2 during health screening procedures, due to environmental exposure risk or household contact with COVID-19 patients. Age, gender, comorbidities, blood groups, clinical and biochemical parameters were recorded, and written informed consents were obtained from all patients prior to the study. Population-level consistency of the blood group distribution in our cohort was compared to the 2024 update from Turkish Red Crescent [https://www.kanver.org/. Accessed 20 Mar 2025]. Severity of the infections were classified as asymptomatic, mild-intermediate or severe (intensive care unit admission) according to the WHO (2020) guidelines [17]. Patient groups were analyzed in two categories: asymptomatic vs. symptomatic (mild-intermediate + severe), and severe vs. non-severe (asymptomatic + mild-intermediate). Further classification was performed according to the number of comorbidities (none, one or greater than one), age intervals (< 35, 35–50, 51–64, ≥ 65) and blood groups (group A vs others).

### Genotyping

DNA isolation from the peripheral blood samples was performed using DNA Blood Kit (Qiagen, Netherlands, Cat. No: 951034) or QIAamp DNA Investigator Kit (Qiagen, Netherlands; Cat. No: 56504) on the Easy1 Advanced XL (Qiagen, Netherlands) platform. KIR and KIR ligand genotyping were performed using Olerup SSP KIR Genotyping (Olerup, Stockholm, Sweden, Cat. No: 104.101-12U) and KIR HLA Ligand PCR SSP (Olerup, Stockholm, Sweden; Cat. No: SSP 104.201-12U) kits, respectively. The resulting PCR amplicons were visualized under UV light after agarose gel electrophoresis to identify KIR-specific amplicon bands. A total of 17 different KIR genes (*KIR2DL1*, *2DL2*, *2DL3*, *2DL4*, *2DL5A*, *2DL5B*, *2DS1*, *2DS2*, *2DS3*, *2DS4*, *2DS5*, *3DL1*, *3DL2*, *3DL3*, *3DS1* and 2 pseudogenes namely *2DP1* and *3DP1*) and 4 KIR ligand genotypes (C1, C2, Bw4, A-Bw4) were analyzed in this study. KIR haplotype motifs were identified based on the KIR gene combinations identified by Cisneros et al. (2020) [18]. For simplicity, the telomeric genotypes tAA + Bw4 + ABw4+ and tAB1 + Bw4 + C2 + are expressed as tAA/wL (with ligands) and tAB1/wL, respectively, throughout the paper. This study included the genotypes tAB1/wL (KIR genes *3DS1* + *2DS5* + *2DS1* + *2DS4* + *2DL5A* + *3DL1* + , and KIR ligands Bw + C2+) and tAA/wL (KIR genes *2DS4* + *3DL1* + , and KIR ligands Bw4 + ABw4+), which were found to be significantly associated with COVID-19 severity in our previous study [5].

Next-generation sequencing was used to analyze extracted DNA samples and identify immune system-associated polymorphisms (rs12252, rs6598045, rs34481144, g.12905756_12905759del and g.12906010)., G > T, G-308A, rs3788979 and rs429358), as well as Neanderthal-derived SNPs in regions 3p21.31 and 12q24.13 (rs11385942, rs10774671, rs1293767, rs1859330, rs1859329 and rs2285932). Either the reference or the alternate alleles of these SNPs have previously been associated with SARS-CoV-2 susceptibility or disease severity in earlier publications (S1 Table). Results were obtained as wild type, heterozygous or homozygous genotypes. *OAS* genotypes were classified as “not homozygous (-) vs homozygous (+)” according to the carriage of at least one Neanderthal-inherited allele in the genotype. According to the reference assembly alleles reported in dbSNP (https://www.ncbi.nlm.nih.gov/snp/. Accessed 31 July 2025), *OAS* genotypes lacking the Neanderthal alleles are annotated as *OAS*1/2/3(+) and genotypes carrying at least one of the Neanderthal-derived protective alleles (rs10774671-G, rs2660-G, rs1293767-C, rs1859330-G, rs1859329-C or rs2285932-T) are annotated as *OAS*1/2/3(-) throughout the paper. SNPs associated with *IFITM3* and *DPP4* were classified based on the presence or absence of the minor alleles in the genotype, i.e., “wild type (-) vs not wild type (+)”, allowing for an exact comparison with the earlier publications. A gene–gene interaction network was illustrated using the GeneMANIA platform (https://genemania.org/. Accessed 23 January 2026) to provide a conceptual overview of known connections between KIR-associated HLA signaling components and other observed genetic associations. Gene variant frequencies derived from Turkish Genome Project (TUSEB) Data Sharing Portal were used for validation of the frequencies across the general Turkish population (https://tgd.tuseb.gov.tr/en/. Accessed 29 Dec 2024). A total of 75 KIR- and HLA-typed donors who had been registered in the Ankara University Donor Registry between 5 February 2013 and 4 December 2020 were also subjected to SNP analysis. These donors had a median age of 43 (25–76) and were included in the study to validate the frequencies of SNPs and KIR/KIR ligands within the Turkish population.

### Statistical analysis

The distribution of KIR genotypes (tAA/wL and tAB1/wL), blood groups, gender and comorbidity frequencies among the asymptomatic, mild-intermediate and severe cases was evaluated using Chi-square or two tailed Fisher’s exact tests as appropriate. For continuous variables, either one-way ANOVA or the Kruskal-Wallis test was used according to the distribution of the variables. In the selection of candidate variables for multivariate analyses comparing asymptomatic vs. symptomatic and severe vs. non-severe patient groups, the significance level was set at 0.10 in univariate tests for each comparison group. According to this criteria, in order to compare asymptomatic and symptomatic groups; age, blood group A, number of comorbidities, tAB1/wL, tAA/wL, *OAS*1/2/3, rs12252 (*IFITM3*), *APOE* ε2ε3 and ε3ε3 genotypes were selected as candidate variables. To compare severe and non-severe groups, age, blood group A, number of comorbidities, tAB1/wL, tAA/wL, *OAS*1/2/3, rs12252 (*IFITM3*), *APOE* ε2ε3 genotype and rs3788979 (*DPP4*) were selected as candidate variables. Multivariate binary logistic regression model was built for both comparisons and analyses were performed by backward elimination method. Area under the ROC curve (AUC) was used to evaluate the discriminative ability of the prediction models. All statistical analyses were performed using IBM SPSS Statistics (version 26; IBM Corporation, Armonk, NY, USA).

## Results

### Clinical and demographic features

The study included 175 unvaccinated individuals with confirmed SARS-CoV-2 infection: 65 were asymptomatic, 47 had mild-to-moderate disease, and 63 experienced severe or critical illness requiring intensive care unit (ICU) admission. Clinical severity was associated with increasing age, male gender, and higher comorbidity burden (Table 1). Blood group A, previously linked to infection susceptibility [19,20], was paradoxically more frequent among asymptomatic individuals in this cohort. Frequency distributions were validated using external controls including 75 donors from Ankara University Donor Registry and data from the Turkish Genome Project Data Sharing Portal (TUSEB) (n = 557) (https://tgd.tuseb.gov.tr/en/. Accessed 29 Dec 2024).

**Table 1:** Patient demographics and frequency distributions across disease severity groups.

Patient Characteristics	All Patients (n=175)			Asymptomatic vs Symptomatic		Non-severe vs Severe	
	Asymptomatic (n=65)	Mild (n=47)	Severe (n=63)	Asymptomatic (n=65)	Symptomatic (n=110)	Non-Severe (n=112)	Severe (n=63)
Age (median [min-max])	35 (18-73)	42 (21-79)	60 (24-94)	35 (18-73)	53 (21-94)	39 (18-79)	60 (24-94)
Age group <35	30 (46.2%)	18 (38.3%)	7 (11.1%)	30 (46.2%)	25 (22.7%)	48 (42.9%)	7 (11.1%)
Age group 35-50	24 (36.9%)	14 (29.8%)	10 (15.9%)	24 (36.9%)	24 (21.8%)	38 (33.9%)	10 (15.9%)
Age group 51-64	9 (13.8%)	12 (25.5%)	19 (30.2%)	9 (13.8%)	31 (28.2%)	21 (18.8%)	19 (30.2%)
Age group ≥65	2 (3.1%)	3 (6.4%)	27 (42.9%)	2 (3.1%)	30 (27.3%)	5 (4.5%)	27 (42.9%)
Gender (Male)	25 (38.5%)	17 (36.2%)	38 (60.3%)	25 (38.5%)	55 (50.0%)	42 (37.5%)	38 (60.3%)
Blood group type A	41 (63.1%)	24 (51.1%)	24 (38.1%)	41 (63.1%)	48 (43.6%)	65 (58.0%)	24 (38.1%)
Blood group type B	9 (13.8%)	8 (17.0%)	10 (15.9%)	9 (13.8%)	18 (16.4%)	17 (15.2%)	10 (15.9%)
Blood group type AB	5 (7.7%)	1 (2.1%)	6 (9.5%)	5 (7.7%)	7 (6.4%)	6 (5.4%)	6 (9.5%)
Blood group type O	10 (15.4%)	14 (29.8%)	23 (36.5%)	10 (15.4%)	37 (33.6%)	24 (21.4%)	23 (36.5%)
Diabetes mellitus	5 (7.7%)	4 (8.5%)	15 (23.8%)	5 (7.7%)	19 (17.3%)	9 (8%)	15 (23.8%)
Hypertension	7 (10.8%)	7 (14.9%)	24 (38.1%)	7 (10.8%)	31 (28.2%)	14 (12.5%)	24 (38.1%)
Chronic obstructive pulmonary disease (COPD)	0 (0%)	0 (0%)	5 (7.9%)	0 (0%)	5 (4.5%)	0 (0%)	5 (7.9%)
Coronary artery disease	2 (3.1%)	2 (4.3%)	4 (6.3%)	2 (3.1%)	6 (5.5%)	4 (3.6%)	4 (6.3%)
Chronic kidney disease	3 (4.6%)	0 (0%)	4 (6.3%)	3 (4.6%)	4 (3.6%)	3 (2.7%)	4 (6.3%)
Cancer	1 (1.5%)	2 (4.3%)	7 (11.1%)	1 (1.5%)	9 (8.2%)	3 (2.7%)	7 (11.1%)
Asthma	0 (0%)	0 (0%)	1 (1.6%)	0 (0%)	1 (0.9%)	0 (0%)	1 (1.6%)
Number of comorbidities (n=0)	54 (83.1%)	36 (76.6%)	27 (42.9%)	54 (83.1%)	63 (57.3%)	90 (80.4%)	27 (42.9%)
Number of comorbidities (n=1)	7 (10.8%)	8 (17.0%)	19 (30.2%)	7 (10.8%)	27 (24.5%)	15 (13.4%)	19 (30.2%)
Number of comorbidities (n≥2)	4 (6.2%)	3 (6.4%)	17 (27.0%)	4 (6.2%)	20 (18.2%)	7 (6.3%)	17 (27.0%)

### KIR motifs and immune variant frequencies

KIR/KIR-ligand motifs tAA/wL and tAB1/wL displayed divergent distributions across disease categories. The tAA/wL motif was enriched in symptomatic cases, while the tAB1/wL motif was more frequent among asymptomatics. Additional genotyping included SNPs in *OAS*1/2/3, *IFITM3*, *DPP4*, *TLR7*, and *APOE* (Table 2). Variants were grouped based on known functional or ancestral profiles. In particular, individuals were classified as *OAS*1/2/3(-) if they carried at least one Neanderthal-derived allele previously shown to increase antiviral *OAS* activity.

**Table 2:** Genotype frequencies of key immunogenetic variants across COVID-19 severity groups.

Genotype	Asymptomatic vs Symptomatic				Non-severe vs Severe			
	Asymptomatic (n=65)	Symptomatic (n=110)	OR (95% CI)	Sig.	Non-severe (n=112)	Severe (n=63)	OR (95% CI)	Sig.
**SNPs within the Neanderthal-haplotype**								
*OAS*1-2-3 (minor alleles)	21 (32.3%)	53 (48.2%)	1.948 (1.027 - 3.696)	0.040	42 (37.5%)	32 (50.8%)	1.720 (0.921 - 3.213)	0.088
**Other COVID-19 associated SNPs**								
*IFITM3* (rs12252-AG/GG)	7 (10.8%)	26 (23.6%)	2.565 (1.044 - 6.303)	0.036	20 (17.9%)	13 (20.6%)	1.196 (0.549 - 2.605)	0.652
*DPP4* (rs3788979-CT/TT)	14 (21.5%)	34 (30.9%)	1.630 (0.796 - 3.336)	0.179	23 (20.5%)	25 (39.7%)	2.546 (1.287 - 5.034)	0.006
***APOE* variants**								
*APOE* (ε2/ε2)	0 (0.0%)	0 (0.0%)	N/A	N/A	0 (0.0%)	0 (0.0%)	N/A	N/A
*APOE* (ε2/ε3)	1 (1.5%)	13 (11.8%)	8.577 (1.095 - 67.183)	0.015	7 (6.3%)	7 (11.1%)	1.875 (0.626 - 5.614)	0.255
*APOE* (ε2/ε4)	0 (0.0%)	1 (0.9%)	N/A	N/A	1 (0.9%)	0 (0.0%)	N/A	N/A
*APOE* (ε3/ε3)	56 (86.2%)	75 (68.2%)	0.344 (0.153 - 0.774)	0.008	84 (75.0%)	47 (74.6%)	0.979 (0.481 - 1.993)	0.954
*APOE* (ε3/ε4)	8 (12.3%)	21 (19.1%)	1.681 (0.698 - 4.051)	0.244	20 (17.9%)	9 (14.3%)	0.767 (0.326 - 1.804)	0.542
KIR (tAA/wL)	4 (6.2%)	21 (19.1%)	3.598 (1.177 - 11.004)	0.018	14 (12.5%)	11 (17.5%)	1.481 (0.628 - 3.493)	0.368
KIR (tAB1/wL)	16 (24.6%)	13 (11.8%)	0.410 (0.183 - 0.921)	0.028	20 (17.9%)	9 (14.3%)	0.767 (0.326 - 1.804)	0.542
*OAS*1-2-3 * tAB1/wL combinations								
*OAS*(+)/tAB1/wL(-)	11 (16.9%)	48 (42.7%)	3.662 (1.729 - 7.756)	<0.001	30 (26.8%)	28 (44.4%)	2.187 (1.142 - 4.187)	0.017
*OAS*(-)/tAB1/wL(+)	6 (9.2%)	7 (6.4%)	0.668 (0.214 - 2.082)	0.485	8 (7.1%)	5 (7.9%)	1.121 (0.350 - 3.584)	0.848
*OAS*(+)/tAB1/wL(+)	10 (15.4%)	6 (5.5%)	0.317 (0.110 - 0.919)	0.028	12 (10.7%)	4 (6.3%)	0.565 (0.174 - 1.832)	0.336
*OAS*(-)/tAB1/wL(-)	38 (58.5%)	50 (45.5%)	0.592 (0.319 - 1.101)	0.096	62 (55.4%)	26 (41.3%)	0.567 (0.303 - 1.059)	0.074

The minor allele of rs12252 (*IFITM3*) was associated with symptomatic disease, and rs3788979 (*DPP4*) with severe disease. The *APOE* ε3ε3 genotype was more common among asymptomatic individuals and appeared protective. Rare *APOE* genotypes (ε2ε2, ε2ε4, ε4ε4) were not observed. A complete list of analyzed variants, with rsIDs, allele designations, and genomic context, is provided in S1 Table.

### KIR-OAS synergy and multivariate analysis

**Fig 1:**
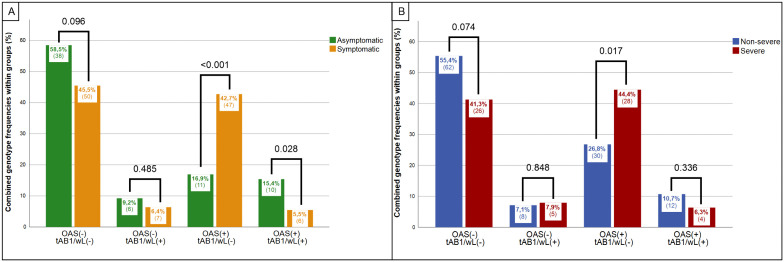
Distribution of *OAS*1/2/3 and tAB1/wL genotype combinations among A) asymptomatic vs. symptomatic and B) non-severe vs. severe patients.

To investigate potential interactions between KIRs and SNPs, frequency distribution of each pair of KIR genotypes and SNPs were compared within the asymptomatic/symptomatic and the non-severe/severe patient groups. Binary Logistic Regression was performed to analyze the individual effects of KIR genotypes, SNPs, and their combined effects. A significant interaction has been found between tAB1/wL and *OAS*1/2/3, favoring their synergistic ability to discriminate between asymptomatic vs. symptomatic groups (P = 0.032). Further analysis revealed that individuals lacking tAB1/wL while carrying *OAS*1/2/3(+) alleles were significantly more likely to be symptomatic (OR 3.471, 95% CI: 1.429–8.432, P = 0.006) and to develop severe disease (OR 2.409, 95% CI: 1.051–5.519, P = 0.038), compared to other genotype combinations (Table 3). This synergistic genotype was significantly rare in asymptomatics but present in over 40% of symptomatic patients (Fig 1).

**Table 3:** Binary logistic regression models identifying independent predictors of the presence of the symptoms and the severity of COVID-19.

Variables included in the final model	Univariate		Multivariate	
	OR (95% CI)	Sig.	OR (95% CI)	Sig.
**Asymptomatic vs. Symptomatic**				
Age (< 35, 35–50, 51–64, ≥ 65)	2.290 (1.628 - 3.223)	0.000	2.673 (1.780 - 4.014)	0.000
Blood type A vs others	0.453 (0.242 - 0.850)	0.014	0.301 (0.136 - 0.669)	0.003
Number of comorbidities (0, 1, ≥ 2)	2.371 (1.393 - 4.036)	0.001		
*IFITM3* (rs12252-AG/GG)	2.565 (1.044 - 6.303)	0.040	4.197 (1.306 - 13.482)	0.016
*APOE* ε2ε3	8.577 (1.095 - 67.183)	0.041		
*APOE* ε3ε3	0.344 (0.153 - 0.774)	0.010	0.189 (0.070 - 0.507)	0.001
tAA/wL (tAA+/Bw4+ABw4+)	3.598 (1.177 - 11.004)	0.025	5.771 (1.635 - 20.370)	0.006
tAB1/wL (tAB1+/C2+ Bw4+)	0.410 (0.183 - 0.921)	0.031		
*OAS*1-2-3	1.948 (1.027 - 3.696)	0.041		
*OAS*1-2-3(+)/tAB1/wL(-)	3.662 (1.729 - 7.756)	0.001	3.471 (1.429 - 8.432)	0.006
**Non-severe vs. Severe**				
Age (< 35, 35–50, 51–64, ≥ 65)	3.296 (2.263 - 4.800)	<0.001	3.422 (2.253 - 5.198)	<0.001
Sex (male)	2.533 (1.345 - 4.772)	0.004	2.543 (1.146 - 5.642)	0.022
Blood type A vs others	0.445 (0.237 - 0.837)	0.012	0.314 (0.137 - 0.722)	0.006
Number of comorbidities (0, 1, ≥ 2)	3.109 (1.946 - 4.968)	<0.001		
*DPP4* (rs3788979-CT/TT)	2.546 (1.287 - 5.034)	0.007	3.995 (1.571 - 10.162)	0.004
tAA/wL (tAA+/Bw4+ABw4+)	1.481 (0.628 - 3.493)	0.370		
tAB1/wL (tAB1+/C2+ Bw4+)	0.767 (0.326 - 1.804)	0.543		
*OAS*1-2-3	1.720 (0.921 - 3.213)	0.089		
*OAS*1-2-3(+)/tAB1/wL(-)	2.187 (1.142 - 4.187)	0.018	2.409 (1.051 - 5.519)	0.038

To crosscheck the independent effects of *OAS* and KIR genotypes and to assess any potential misleading effects due to collinearity, frequency distributions were analyzed across the entire cohort, including both patients and registry population. Cross-tabulation of *OAS*1/2/3 with tAB1/wL or tAA/wL genotypes, regardless of the study groups, revealed no significant differences in their frequencies, suggesting that the associations with disease progression are not due to the uneven distribution of the genotypes (S2 Table).

**Fig 2:**
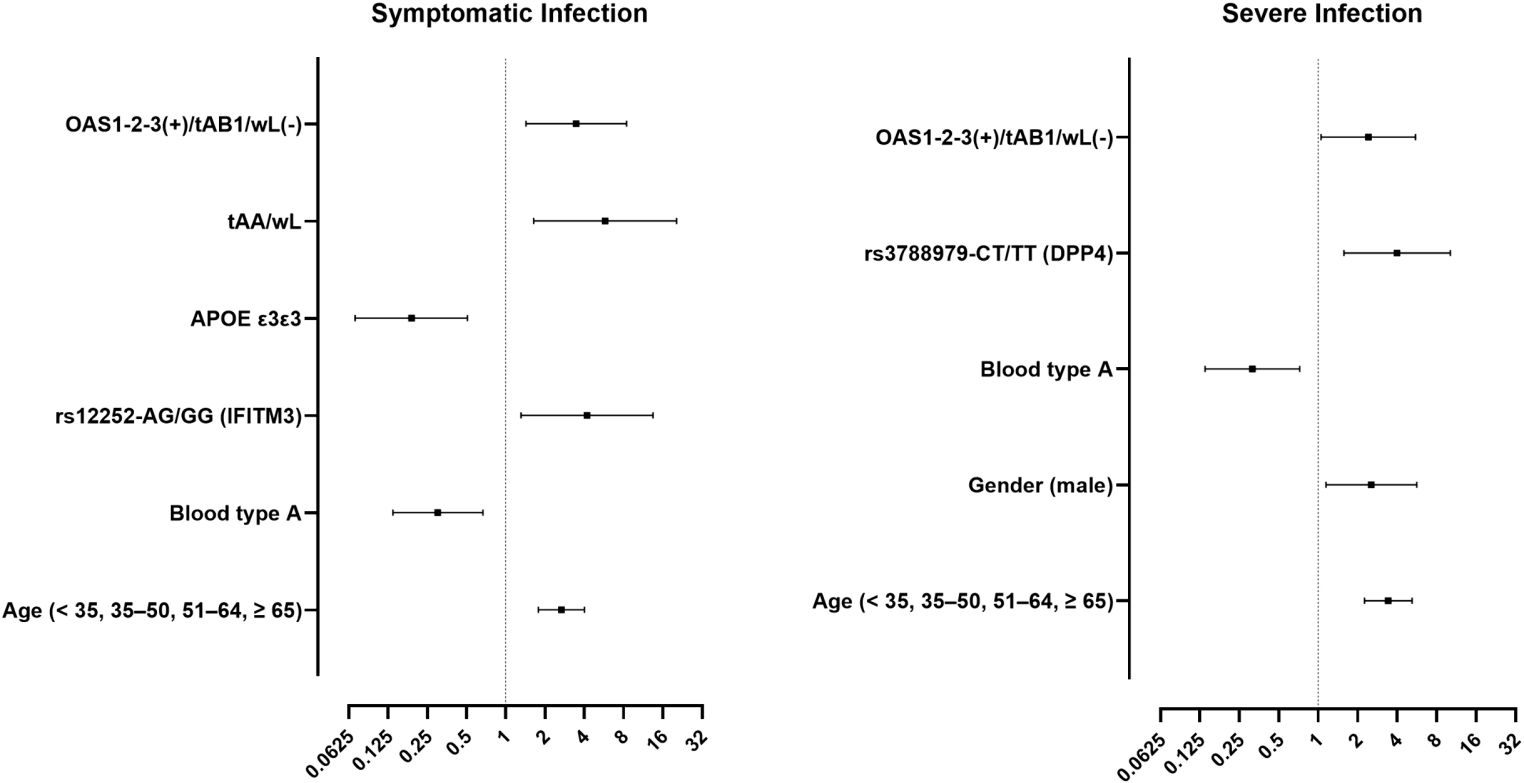
Forest plots showing odds ratios and 95% confidence intervals for predictors of symptomatic (left) and severe (right) COVID-19.

Multivariate logistic regression identified age, blood group A, *IFITM3* rs12252, *APOE* ε3ε3, tAA/wL status, and the *OAS*1/2/3(+)/tAB1/wL(-) interaction as independent predictors of symptomatic infection. For severe disease, the final model retained age, male gender, blood group A, *DPP4* rs3788979, and the same synergistic *OAS*1/2/3(+)/tAB1/wL(-) genotype (Table 3, Fig 2).

Model performance was robust: The symptomatic vs. asymptomatic classifier achieved 75.4% accuracy with an AUC of 0.849, sensitivity of 81.4%, specificity of 69.2%, and a positive predictive value (PPV) of 76.6%. The severe vs. non-severe model achieved 78.9% accuracy with an AUC of 0.861, sensitivity of 79.4%, specificity of 78.4%, and a PPV of 73.9%. Full performance metrics are provided in S1 Fig.

### External controls and rare variant analysis

External controls consisting of healthy donors and publicly available reference data from the Turkish Genome Project (TUSEB), which is a national whole-genome reference repository (https://tgd.tuseb.gov.tr/en/. Accessed 29 Dec 2024), were used to validate the accurate representation of population-specific genotype frequencies within the patient cohort. No carriers of rare *TLR7* missense or loss-of-function variants were detected in any of the study groups. *APOE* genotypes were classified based on ε2, ε3 and ε4 allele combinations (ε2ε2, ε2ε3, ε2ε4, ε3ε3, ε3ε4, ε4ε4). Homozygosity for ε2 and ε4 alleles was not observed in any of the patients. Only two cases from the donor registry were identified as ε2 homozygous. Full *APOE* genotype distributions and comparative frequencies are presented in S3 Table.

## Discussion

**Fig 3:**
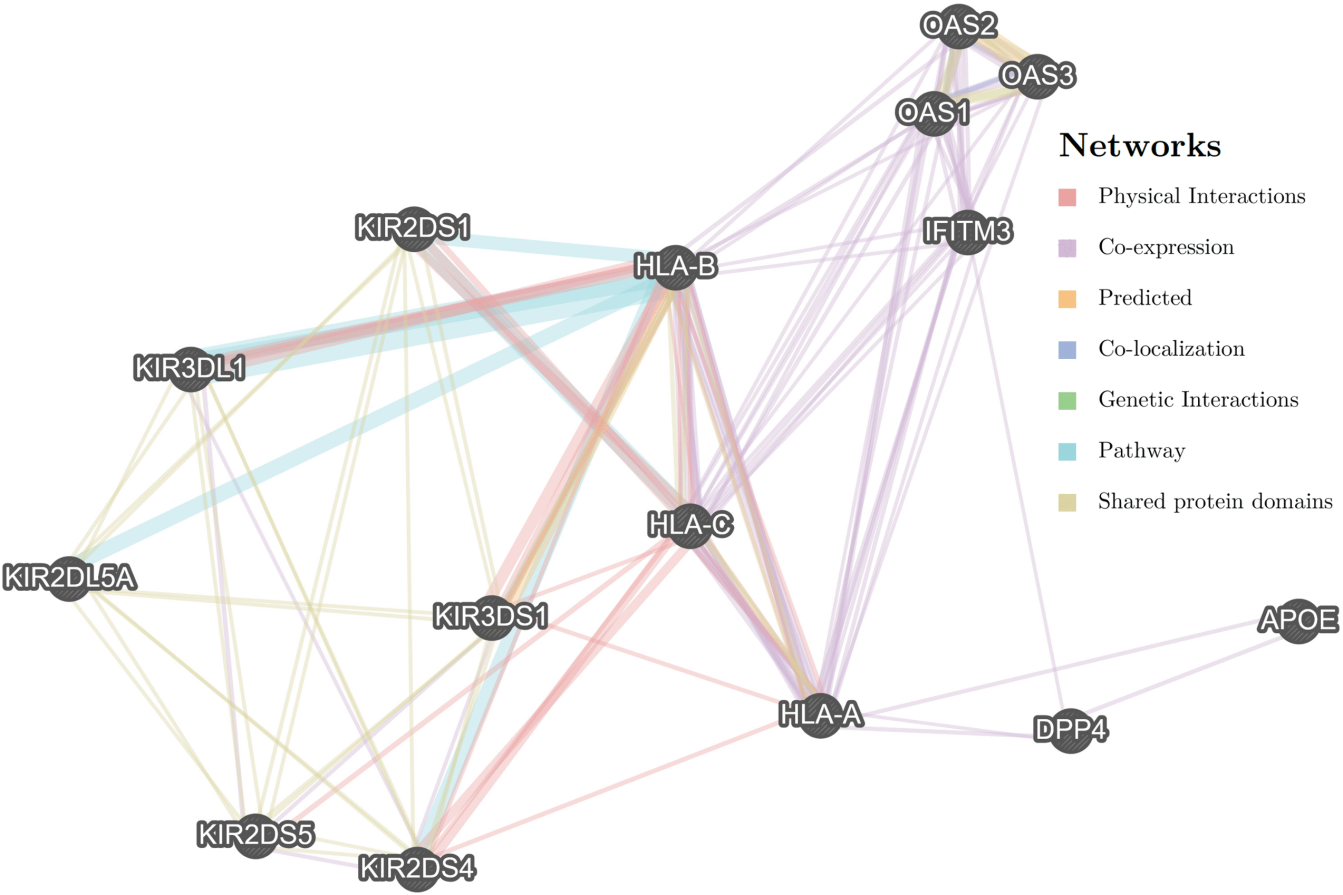
Gene–gene interaction network generated using the GeneMANIA platform illustrating known functional, physical, and pathway-based relationships among genes investigated in this study [21]. The network highlights interactions linking KIR-associated HLA signaling with interferon-stimulated antiviral pathways (*OAS1*, *OAS2*, *OAS3*, *IFITM3*, *TLR7*) and immunometabolic regulators (*APOE*, *DPP4*). Edges represent previously reported co-expression, physical interaction, pathway co-membership, or shared protein domains.

Our study demonstrates, for the first time, a synergistic effect between the absence of the tAB1/wL KIR motif and the presence of Neanderthal-derived *OAS* variants [*OAS*1/2/3(-)] in predicting both symptomatic and severe COVID-19 progression. Age, male gender, blood group A, and SNPs in *IFITM3* and *DPP4* further contributed to disease outcomes. These findings advance the understanding of how both recent immune gene evolution (KIR polymorphisms) and ancient introgressed variants (*OAS* haplotypes) jointly modulate human viral susceptibility. To provide a biological framework for the observed genetic associations, we generated a gene–gene interaction network using the GeneMANIA platform [21]. This network illustrates known connections between KIR-associated HLA signaling, interferon-mediated antiviral pathways, and immunometabolic regulators, supporting the plausibility of gene–gene interactions underlying COVID-19 symptomatology and severity and is presented as a conceptual framework to provide biological context for the observed genetic associations rather than as direct evidence derived from the present cohort (Fig 3).

In this study, we evaluated a panel of immune-related SNPs previously associated with COVID-19 severity and mortality, rather than conducting an untargeted genome-wide analysis. By assessing these variants across clinically defined categories – asymptomatic vs symptomatic and non-severe vs severe – we aimed to contextualize their individual contributions to disease progression.

Our findings suggest a layered genetic influence along the symptomatic-to-severe disease spectrum. *IFITM3* rs12252-GG and *APOE* ε3ε3 genotype were associated primarily with the presence and absence of symptoms, respectively; while *DPP4* rs3788979-TT was more strongly linked to progression toward severe disease. These findings are consistent with the proposed roles of these gene polymorphisms in the course of infection. While the *IFITM3*-GG genotype has been associated with impaired viral restriction [13], *APOE* ε4 variants have been linked increased cholesterol levels and enhanced accumulation of ACE2 and TMPRSS2 within cholesterol-enriched domains [22]. Although these factors alone are insufficient to explain the entire mechanism, it is reasonable to suggest that patients lacking *APOE* ε3ε3 genotype and carrying *IFITM3*-GG variants may be more prone to a symptomatic course of infection, as host immunity may fail to effectively prevent viral entry and replication. *DPP4*, on the other hand, is suggested to play multiple roles in COVID-19 pathogenesis [14]. In addition to its involvement in viral entry, its physiological effects on T cell activation, cell adhesion and apoptosis may exert a greater influence on disease severity rather than on symptomatic determination. Although rare deleterious *TLR7* variants have been associated with severe COVID-19 in young males, their absence in our cohort is consistent with their very low population frequency and the limited sample size. Notably, the interaction between *OAS*1/2/3(+) and absence of the protective tAB1/wL KIR motif was a shared predictor of both symptom onset and severity, suggesting it may represent a broader axis of innate immune dysregulation relevant throughout the clinical course. Moreover, cross-sectional frequency distributions of *OAS*1/2/3 and tAB1/wL combinations among the significant predictors of symptomatic and severe disease confirm that the predisposing role of *OAS*1/2/3(+)/tAB1/wL(-) genotype was preserved despite the lack of additional risk factors, i.e., older age, male gender, *IFITM3* rs12252, or *DPP4* rs3788979 status (S2 Fig).

While some variants may plausibly influence both stages of disease (e.g., *IFITM3* or *DPP4*), the lack of statistical significance in one of the comparisons may reflect power limitations rather than true absence of effect. These observations support the hypothesis that distinct genetic mechanisms may underlie the transition from viral containment (asymptomatic vs symptomatic) and from immune control failure (non-severe vs severe), with partially overlapping yet non-identical contributors.

Consistent with prior studies [23], advanced age was the strongest predictor of severity. Comorbidity burden correlated with severity in univariate models but was not retained in multivariate models, likely reflecting cohort size limitations. While blood group A has been associated with infection susceptibility [19,20], there have been contradictory conclusions in worldwide studies regarding its association with disease severity [4]. The distribution of blood groups in our cohort is consistent with the earlier reports and the 2024 update from Turkish Red Crescent [24,20]; nevertheless, the protective role of blood group A against symptomatic and severe COVID-19 observed in our study is novel within Turkish populations and warrants further study in larger populations.

Our findings align with studies showing inhibitory KIR motifs (e.g., tAA) predispose to worse outcomes, whereas activating motifs (e.g., tAB1) are protective [6,25]. In contrast to previous studies examining individual KIR genes or general haplotype groups, such as the study by Littera et al. (2021) [6], our analysis focused on composite KIR genotypes defined by specific activating or inhibitory motifs (tAA and tAB1) in combination with their respective HLA ligands. This approach was chosen to reduce dimensionality and preserve statistical power in the context of a limited sample size. Moreover, evaluating ligand-associated KIR motifs allowed us to target broader NK cell regulatory effects, rather than isolated gene-level signals. While this genotypic definition increases specificity, it may reduce generalizability and comparability with studies using broader haplotype categories. Therefore, rather than testing all possible motif combinations, we focused on genotypes previously associated with clinical outcomes in our earlier cohort [5]. Differences in KIR association results across studies likely reflect ethnic-specific KIR-HLA diversity and variable analysis strategies (allelic vs haplotypic).

Supporting Zeberg and Pääbo (2021), carriers of Neanderthal-protective *OAS*1/2/3 alleles exhibited reduced risk of symptomatic or severe infection [9]. However, this protection was overcome when combined with absence of the protective tAB1/wL genotype – highlighting gene-gene interactions in innate antiviral defense.

The rs12252 variant in *IFITM3* and the rs3788979 variant in *DPP4* were associated with symptomatic infection and disease severity, respectively, consistent with prior meta-analyses [14,22]. *APOE* ε3ε3 conferred relative protection, though associations involving rare *APOE* variants (e.g., ε2ε3) require cautious interpretation due to small sample sizes. No rare *TLR7* loss of function variants were detected, consistent with their extremely low frequency; larger male-enriched cohorts would be needed for detection.

From the evolutionary perspective, the interaction between Neanderthal *OAS* alleles and KIR genotypes supports the concept that introgressed innate immune alleles and recent KIR diversification both shaped modern human responses to RNA viruses. Such dual-layered genetic architecture likely conferred historical survival advantages but now influences modern disease susceptibilities.

This study has several notable strengths. By integrating KIR haplotype motifs, Neanderthal-inherited *OAS*1/2/3 variants, and modern immune-related SNPs within the same predictive models, we provide a comprehensive immunogenetic evaluation of COVID-19 outcomes. The use of a homogenous population, validated against national genomic databases, minimizes the risk of population stratification bias. Furthermore, the application of multivariate logistic regression, ROC curve analysis, and cross-validation with healthy donor controls enhances the robustness of our findings.

Earlier, the population-based distributions of Neanderthal-inherited SNPs or KIR genes have been published [9,18,26]. Likewise, susceptibility against COVID-19 has also been shown to be influenced by ethnic background. In our earlier study, KIR genotype frequencies in our geographical region were similar to those of Europeans [5]. According to Zeberg and Pääbo (2021), frequencies of Neanderthal-introgressed *OAS* haplotypes have increased over time, i.e., *OAS*3 rs1156361 from below 10% to ~30%, in Eurasia in the last 20,000 years [9]. In this study, Neanderthal-inherited allele frequency of *OAS* SNPs was ~ 57% in the registry donors, similar to that in the TUSEB (https://tgd.tuseb.gov.tr/en/. Accessed 29 Dec 2024), consistent with the idea of positive selection of innate immunity gene variants in modern humans.

Nevertheless, some limitations should be acknowledged. First, our genotyping approach did not resolve KIR copy number variation, which could contribute additional phenotypic variability. Second, the relatively small sample size limited our power to detect associations involving rare genetic variants, particularly in *APOE* and *TLR7*. Third, while population homogeneity strengthens internal validity, it also restricts the generalizability of our results to broader, multiethnic populations. Finally, by combining mild-intermediate and severe cases into a single “symptomatic” group for some analyses, we may have partially obscured biological differences between truly mild and critically severe disease trajectories. These limitations notwithstanding, the observed synergy between KIR and *OAS*1/2/3 genotypes offers novel insights into the layered evolutionary architecture of innate antiviral immunity.

## Conclusions

In this study, we investigated potential evolutionary interactions among the multifactorial components of immune defense by extending our previous findings on KIR - COVID-19 associations. We uncovered a novel immunogenetic interaction between KIR motifs and Neanderthal-derived *OAS*1/2/3 variants impacting COVID-19 symptomatology and severity. The validity of our findings may differ in diverse populations which require further investigation, incorporate deeper KIR characterization (including CNV), and explore functional consequences of these gene–gene interactions in antiviral immunity. After validation in independent cohorts, these immunogenetic signatures may contribute to genetic risk stratification and, in selected settings, inform personalized preventive or monitoring strategies for susceptible individuals.

## Supporting information

S1 Table Summary of SNPs analyzed, including rsIDs, genomic context, and known risk allele annotations. (PDF)

S2 Table Frequency distribution of OAS1/2/3 and KIR genotypes across the full study cohort (patients + registry donors, n = 250). (PDF)

S1 Fig ROC curves for models predicting A) symptomatic and B) severe COVID-19, with (red) and without (blue) inclusion of the OAS1/2/3(+)/tAB1wL(-) genotype. (PDF)

S3 Table Genotype frequencies in COVID-19 patients and comparative populations, including data from the Turkish Genome Project (TUSEB). (PDF)

S2 Fig Cross-stratified distribution of OAS1/2/3*tAB1/wL genotypes among individuals with or without other significant predictors of symptomatic and severe disease. (PDF)
