# Supplementary material for: Innate immunogenetic synergy between KIR and Neanderthal-derived OAS variants predicts COVID-19 outcomes
Source: PLoS One. 2026 May 27;21(5):e0345137. doi: 10.1371/journal.pone.0345137 (PMC13215513; doi:10.1371/journal.pone.0345137)
Supplement: S1 Table — (PDF) [file pone.0345137.s001.pdf]

Supplementary Table 1. Summary of SNPs analyzed, including rsIDs, genomic context, and known risk allele annotations.

**Chromosomal Location**	**Reference SNP ID**	**Genes Affected**	**COVID-19 Associated Risk Allele**
12q24.13	***OAS1*:**<br>rs10774671 G>A<br>***OAS2*:**<br>rs1293767 C>G<br>***OAS3*:**<br>rs1859330 G>A<br>rs1859329 C>T<br>rs2285932 T>C<br>rs2660 G>A	*OAS1, OAS2, OAS3*	Reference alleles of all SNPs
11p15.5	rs12252 A>G	*IFITM3*	Alternate (rs12252-G)
Xp22.2	rs2042915990 AACT>-<br>rs200553089 G>T	*TLR7*	Alternate (rs2042915990-del)
2q24.3	rs3788979 C>T	*DPP4*	Alternate (rs3788979-T)
19q13.32	rs429358 T>C and rs7412 C>T	*APOE* (ε2ε2, ε2ε3, ε2ε4, ε3ε3, ε3ε4, ε4ε4)	*APOE* ε4
