# Supplementary material for: Innate immunogenetic synergy between KIR and Neanderthal-derived OAS variants predicts COVID-19 outcomes
Source: PLoS One. 2026 May 27;21(5):e0345137. doi: 10.1371/journal.pone.0345137 (PMC13215513; doi:10.1371/journal.pone.0345137)
Supplement: S2 Table — (PDF) [file pone.0345137.s002.pdf]

Supplementary Table 2. Frequency distribution of *OAS*1/2/3 and KIR genotypes across the full study cohort (patients + registry donors, n=250).

Frequency Distribution of *OAS*1/2/3 and KIR genotypes n (%)	*OAS*1/2/3 negative	*OAS*1/2/3 **positive**	*OAS* 1/2/3 total	P value
tAB1/wL negative	117 (46,8%)	79 (31,6%)	196 (78,4%)	0.202
tAB1/wL **positive**	27 (**10,8%**)	27 (**10,8%**)	54 (21,6%)	
tAB1/wL total	144 (57,6%)	106 (42,4%)	250 (100%)	
tAA/wL negative	119 (47,6%)	82 (32,8%)	201 (80,4%)	0.299
tAA/wL **positive**	25 (**10,0%**)	24 (**9,6%**)	49 (19,6%)	
tAA/wL total	144 (57,6%)	106 (42,4%)	250 (100%)	
