# Supplementary material for: Innate immunogenetic synergy between KIR and Neanderthal-derived OAS variants predicts COVID-19 outcomes
Source: PLoS One. 2026 May 27;21(5):e0345137. doi: 10.1371/journal.pone.0345137 (PMC13215513; doi:10.1371/journal.pone.0345137)
Supplement: S1 Fig — (PDF) [file pone.0345137.s003.pdf]

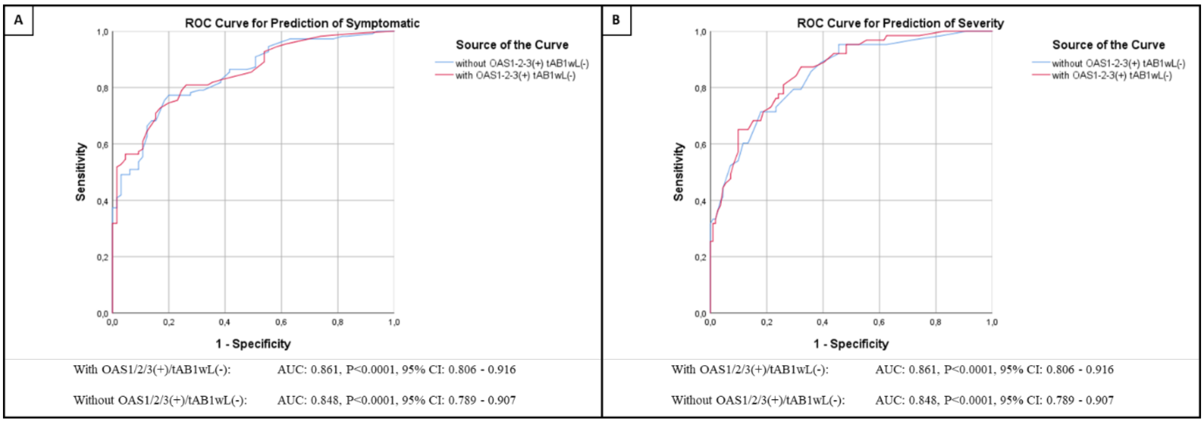

Supplementary Figure 1. ROC curves for models predicting A) symptomatic and B) severe COVID-19, with (red) and without (blue) inclusion of the *OAS*1/2/3(+)/tAB1wL(-) genotype.
