# Supplementary material for: Innate immunogenetic synergy between KIR and Neanderthal-derived OAS variants predicts COVID-19 outcomes
Source: PLoS One. 2026 May 27;21(5):e0345137. doi: 10.1371/journal.pone.0345137 (PMC13215513; doi:10.1371/journal.pone.0345137)
Supplement: S3 Table — (PDF) [file pone.0345137.s004.pdf]

Supplementary Table 3. Genotype frequencies in COVID-19 patients and comparative populations, including data from the Turkish Genome Project (TUSEB).

**Genotype**	**TUSEB (n=557)**	**Registry Donors (n=75)**	**Patients (n=175)**		
			**Asymptomatic (n=65)**	**Mild (n=47)**	**Severe (n=63)**
**SNPs from Neanderthal-inherited loci**					
*OAS*1 (rs10774671-AA)	29 (38.2%)	26 (34.7%)	18 (27.7%)	19 (40.4%)	29 (46%)
*OAS*1 (rs2660-AA)	31 (41.5%)	26 (34.7%)	18 (27.7%)	16 (34.0%)	29 (46%)
*OAS*2 (rs1293767-GG)	34 (45.8%)	32 (42.7%)	19 (29.2%)	21 (44.7%)	30 (47.6%)
*OAS*3 (rs1859330-AA)	31 (41.1%)	26 (34.7%)	18 (27.7%)	20 (42.6%)	29 (46%)
*OAS*3 (rs1859329-TT)	328 (42.0%)	26 (34.7%)	18 (27.7%)	20 (42.6%)	29 (46%)
*OAS*3 (rs2285932-CC)	31 (41.1%)	31 (41.3%)	21 (32.3%)	20 (42.6%)	31 (49.2%)
*OAS*1-2-3	N/A	32 (42.7%)	21 (32.3%)	21 (44.7%)	32 (50.8%)
**Other COVID-19 associated SNPs**					
IFITM3 (rs12252-AG/GG)	94 (16.9%)	14 (18.7%)	7 (10.8%)	13 (27.7%)	13 (20.6%)
DPP4 (rs3788979-CT/TT)	18 (23.9%)	19 (25.3%)	14 (21.5%)	9 (19.1%)	25 (39.7%)
***APOE* variants**					
APOE ε2/ε2	N/A	2 (2.7%)	0 (0.0%)	0 (0.0%)	0 (0.0%)
APOE ε2/ε3	N/A	11 (14.7%)	1 (1.5%)	6 (12.8%)	7 (11.1%)
APOE ε2/ε4	N/A	1 (1.3%)	0 (0.0%)	1 (2.1%)	0 (0.0%)
APOE ε3/ε3	N/A	54 (72.0%)	56 (86.2%)	28 (59.6%)	47 (74.6%)
APOE ε3/ε4	N/A	7 (9.3%)	8 (12.3%)	12 (25.5%)	9 (14.3%)
KIR tAA/wL	N/A	24 (32.0%)	4 (6.2%)	10 (21.3%)	11 (17.5%)
KIR tAB1/wL	N/A	25 (33.3%)	16 (24.6%)	4 (8.5%)	9 (14.3%)
***OAS*1-2-3 * tAB1/wL combinations**					
OAS(+) tAB1/wL(-)	N/A	21 (28.0%)	11 (16.9%)	19 (40.4%)	28 (44.4%)
OAS(-) tAB1/wL(+)	N/A	14 (18.7%)	6 (9.2%)	2 (4.3%)	5 (7.9%)
OAS(+) tAB1/wL(+)	N/A	11 (14.7%)	10 (15.4%)	2 (4.3%)	4 (6.3%)
OAS(-) tAB1/wL(-)	N/A	29 (38.7%)	38 (58.5%)	24 (51.1%)	26 (41.3%)
