# Supplementary material for: Innate immunogenetic synergy between KIR and Neanderthal-derived OAS variants predicts COVID-19 outcomes
Source: PLoS One. 2026 May 27;21(5):e0345137. doi: 10.1371/journal.pone.0345137 (PMC13215513; doi:10.1371/journal.pone.0345137)
Supplement: S2 Fig — (PDF) [file pone.0345137.s005.pdf]

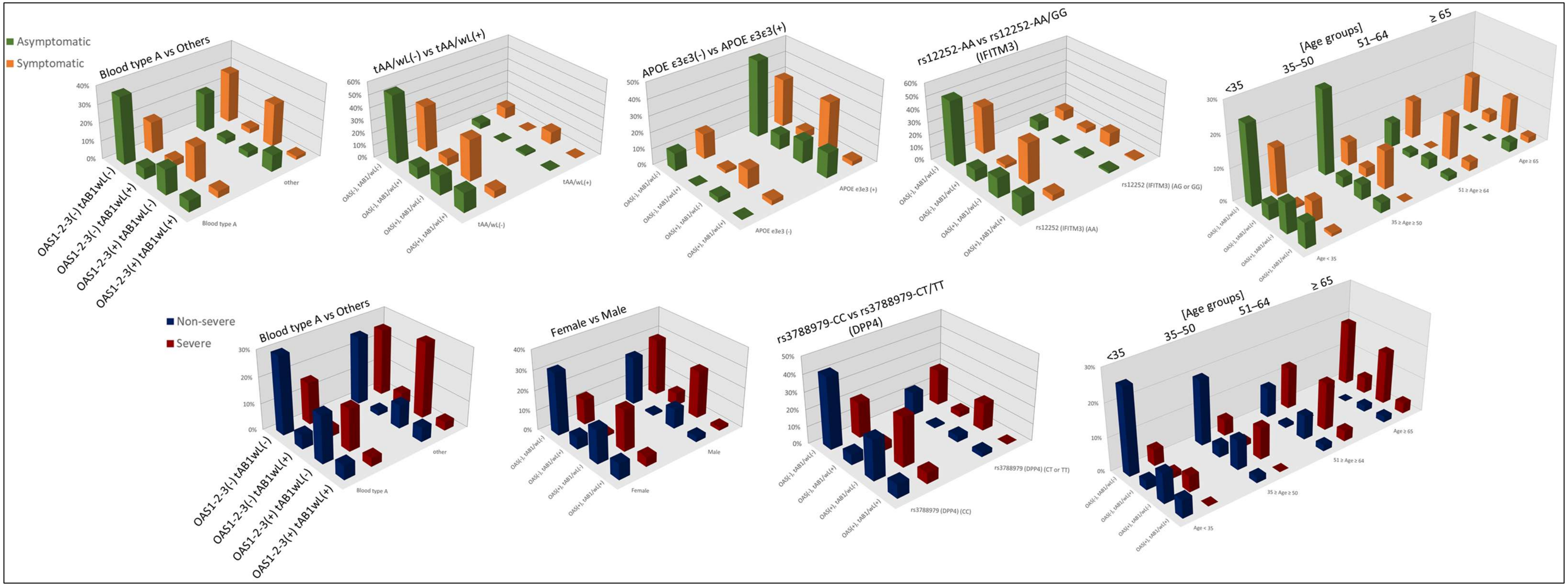


**Supplementary Figure 2.** Cross-stratified distribution of OAS1/2/3*tAB1/wL genotypes among individuals with or without other significant predictors of symptomatic and severe disease.
